# Research Progress on Acidizing Techniques for Complex-Type Reservoirs

**DOI:** 10.3390/molecules31183184

**Published:** 2026-09-10

**Authors:** Yujie Bai, Yifei Sun, Chao Xu, Mingxing Bai, Jiashu Wu, Jingfang Cui, Guangsheng Cao, Gen Li

**Affiliations:** 1 Key Laboratory of Enhanced Oil and Gas Recovery of Ministry of Education, Northeast Petroleum University, Daqing 163318, China; 2 Production Operation Department, Xinjiang Oilfield Company, PetroChina, Karamay 834000, China; 3 Department of Mathematics, College of Sciences, Northeastern University (Nanhu Campus), Shenyang 110819, China

**Keywords:** acidizing technology, complex reservoirs, molecular technology, reservoir stimulation, enhanced oil recovery

## Abstract

With the continuous decline in easily recoverable reserves of conventional oil and gas, the focus of oil and gas exploration and development has gradually shifted toward complex reservoirs, such as low-permeability, tight, high-temperature and high-pressure (HTHP), and strongly heterogeneous reservoirs. Such reservoirs are characterized by complex pore–throat structures, poor seepage capacity, extreme temperature and pressure conditions, and strong heterogeneity. Traditional acidizing technologies face multiple challenges, including short effective penetration distances, system instability at high temperatures, uneven stimulation, and a tendency to induce secondary formation damage, making it difficult to meet the requirements for highly efficient reservoir stimulation. This paper systematically analyzes the petrophysical properties and acidizing challenges of four typical types of complex reservoirs. It reviews the reaction mechanisms, research and development progress, and reservoir adaptability principles of six acid systems, and elaborates on the application value of molecular simulation technology in acid–rock reaction analysis, formulation optimization, and injection regulation. The review indicates that, under extreme operating conditions, existing technologies still suffer from inadequate system stability, limited deep mass transfer, and environmental concerns. Future research should deepen the understanding of multi-scale acid–rock reaction mechanisms, develop composite acid systems tolerant to extreme conditions, and drive the development of acidizing technologies toward refinement and intelligentization.

## 1. Introduction

As the development of conventional medium- to high-permeability oil and gas reservoirs enters the middle and late stages, easily recoverable reserves continue to decline, and oil and gas exploration and development are continuously extending to complex types of reservoirs such as deep, ultra-deep, low-porosity and low-permeability, strongly heterogeneous, and fractured-vuggy reservoirs [1]. Influenced jointly by sedimentary environments, diagenesis, and tectonic modifications, such reservoirs generally exhibit characteristics such as complex pore–throat structures, poor seepage capacity, strong reservoir sensitivity, extreme temperature and pressure conditions, and large differences in physical properties between layers [2]. Their natural productivity is relatively low, and their stable production capacity is insufficient; thus, conventional development and stimulation methods make it difficult to achieve the efficient utilization of reserves [3].

As the core stimulation technology for increasing production and injection in oil and gas reservoirs, acidizing effectively improves reservoir seepage conditions by injecting functional acid systems into the near-wellbore formation and utilizing acid–rock chemical reactions to dissolve wellbore damage, clear pore throats, and connect natural fractures and fracture-vuggy networks. It has been widely applied in various types of oil and gas fields [4]. However, in the face of complex reservoirs with harsh geological conditions, the acid–rock reaction rate is difficult to control. Acid fluids are mostly consumed rapidly in the near-wellbore zone, resulting in a limited effective penetration distance. Under high-temperature and high-pressure environments, acid fluids and additives are prone to degradation, demulsification, and viscosity reduction, making it difficult to guarantee system stability. In strongly heterogeneous formations, acid fluids easily channel along high-permeability streaks, leaving low-permeability enriched zones inadequately stimulated and causing unbalanced overall reservoir utilization. Meanwhile, the retention of spent acid, hydration and swelling of clay minerals, and fines migration after acidizing can easily induce secondary formation damage, further restricting the long-term effectiveness of acidizing stimulation [5,6].

To meet the stimulation requirements of complex reservoirs, researchers and oilfield technicians at home and abroad have conducted extensive laboratory experiments and field practices focusing on acid formulation modification, action mechanism elucidation, operation process optimization, and field adaptability evaluation. They have successively developed various new acid systems, such as retarded, temporarily plugging, diverting, temperature-resistant, and low-damage acids, and formulated supporting technological processes, including zonal acidizing, temporary plugging and diverting acidizing, and combined acid-fracturing stimulation, significantly enriching the acidizing stimulation technology system for complex reservoirs [7,8]. Among these, molecular technology is widely applied in understanding action mechanisms, formulation design, and injection parameter optimization.

Currently, existing studies mostly focus on the optimization of a single acid formulation, the stimulation effect of a certain type of reservoir, or the application summary of a single process, lacking a systematic review and summary from an overall perspective on the acidizing difficulties of complex reservoirs, the development trajectory of various acid fluid technologies, application bottlenecks, and future research directions [9].

The methodologies applied in reservoir acidizing diverge significantly depending on the target rock mineralogy, fundamentally divided into carbonate and sandstone approaches. In carbonate reservoirs (predominantly composed of CaCO_3_ and CaMg(CO_3_)_2_), the methodology focuses on macroscopic matrix dissolution. Standard treatments primarily utilize hydrochloric acid (HCl) to aggressively react with the soluble rock matrix. The objective is to harness flow-reaction coupling to create highly conductive, macroscopic flow channels known as wormholes, which extend deep into the formation to bypass near-wellbore damage. Conversely, sandstone reservoirs, composed of chemically stable quartz, require a distinctly different methodology. The treatment relies on mud acid—a synergistic mixture of hydrofluoric (HF) and hydrochloric (HCl) acids. Rather than dissolving the bulk rock structure (which could induce formation collapse), HF is specifically targeted to dissolve pore-plugging siliceous clays, fines, and feldspars. Concurrently, HCl is essential, often as a pre-flush, to remove authigenic carbonate cements and prevent the secondary precipitation of insoluble fluosilicates. Consequently, while carbonate acidizing aims to construct new macro-conduits, sandstone acidizing focuses strictly on meticulously restoring the original microscopic permeability without compromising rock mechanics [10,11].

**Figure 1:**
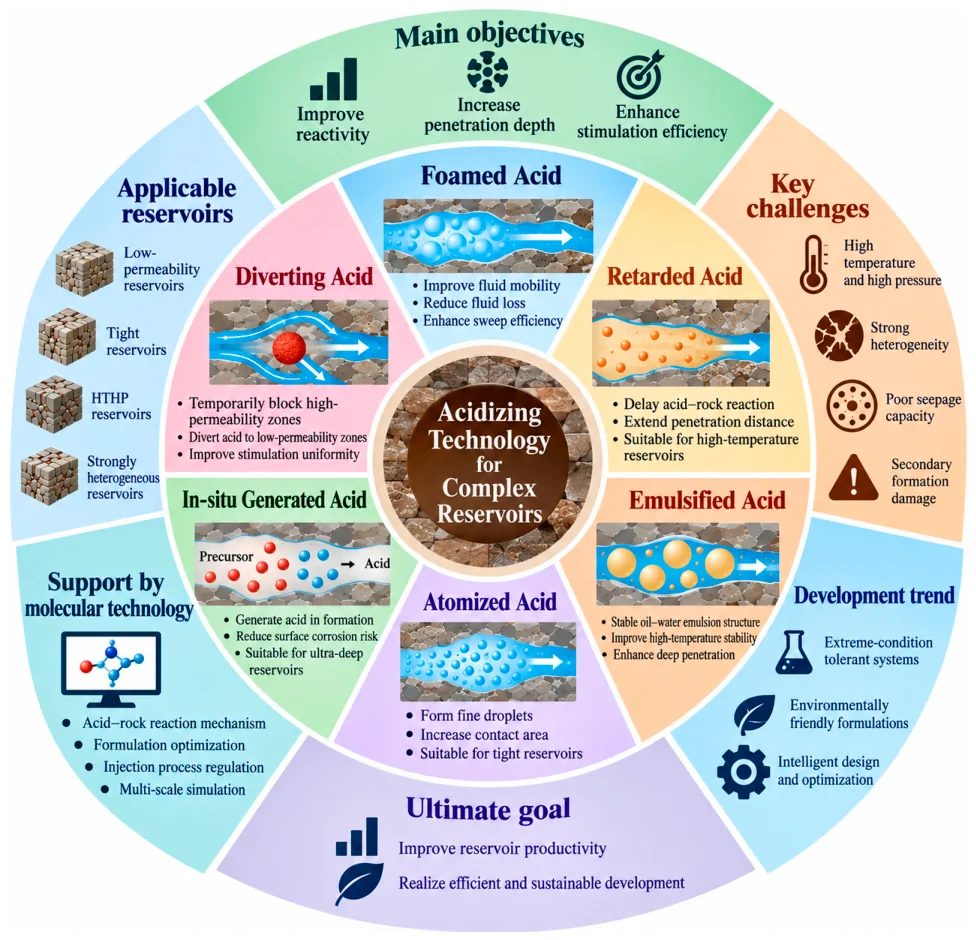
Overview of complex reservoir types and their acidizing techniques.

As shown in Figure 1, this figure summarizes the complex reservoirs discussed in this paper along with the corresponding technologies. Based on the research achievements of existing domestic and foreign literature, this paper comprehensively analyzes the geological characteristics and common acidizing challenges of complex reservoirs, reviews the action mechanisms, research progress, and field application status of mainstream acidizing working fluid systems, and analyzes the application status and direction of molecular technology in reservoir acidizing. According to the development trend of oil and gas exploration and development, combined with policy directions, the development path of acidizing technology is analyzed, aiming to provide a theoretical reference for the selection of acidizing formulations, process design, and large-scale field application in complex oil and gas reservoirs.

## 2. Complex Reservoirs and Development Characteristics

### 2.1. Complex Reservoirs

**Figure 2:**
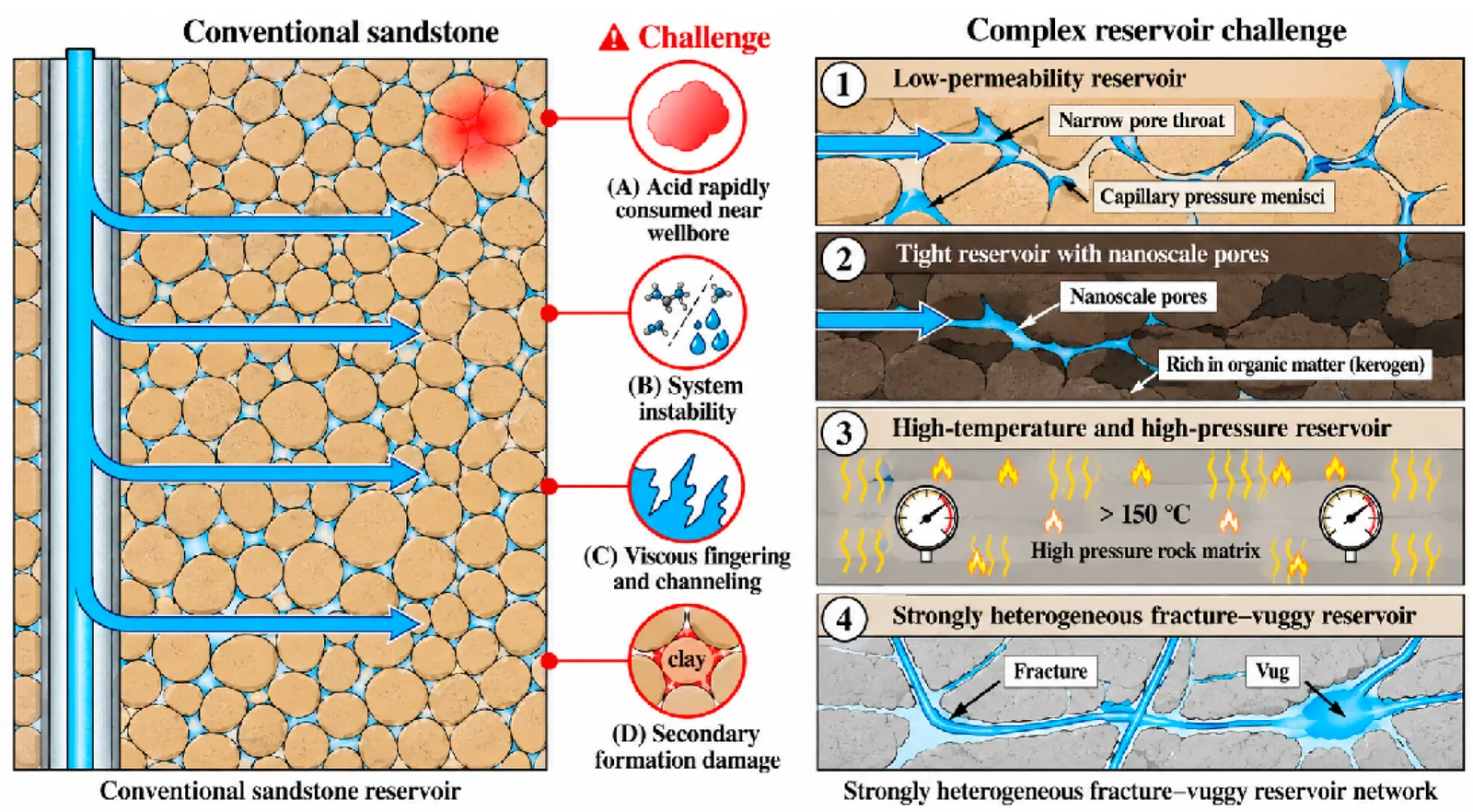
Four major classifications and characteristics of complex reservoirs.

Currently, complex reservoirs are important targets for oil and gas exploration and development. Their development is highly difficult, and acidizing technologies face the challenges of insufficient reservoir adaptability and long-term stimulation effectiveness [12]. Based on their geological characteristics and engineering parameters, this paper classifies complex reservoirs into four major categories: low-permeability reservoirs, tight reservoirs, high-temperature and high-pressure reservoirs, and strongly heterogeneous reservoirs. Each type of reservoir has unique physical property characteristics and acidizing difficulties [13], as shown in Figure 2.

(1) Low-permeability reservoirs

Low-permeability reservoirs are defined in this review as reservoirs with permeability ranging from 0.1 to 50 mD. Based on the permeability range, they can be further subdivided into ultra-low-permeability reservoirs (0.1–1 mD), extra-low-permeability reservoirs (1–10 mD), and low-permeability reservoirs (10–50 mD) [14]. These reservoirs typically exhibit small pore–throat radii, large specific surface areas, and strong capillary effects. In such reservoirs, capillary pressure, which is governed by pore–throat size, interfacial tension, and rock wettability, significantly influences acid-fluid entry and distribution within the pore network. This does not imply that acid flows exclusively through water-wet channels; rather, wettability affects the magnitude and direction of capillary forces and consequently the preferential distribution of the acid fluid. In addition, an unfavorable mobility or viscosity contrast between the injected acid and the displaced reservoir fluid may lead to viscous fingering, resulting in uneven acid distribution and non-uniform acid–rock reactions [15]. The limited fluid-flow capacity also makes spent-acid flowback difficult. Retained spent acid may induce the swelling, dispersion, and migration of clay minerals, ultimately causing pore–throat plugging and secondary formation damage [16]. Studies have shown that maintaining a viscosity ratio of 100:1–150:1 can effectively suppress viscous fingering and improve acid sweep efficiency [17,18]. A representative example is the Bai 823 well area, where low-permeability sandstone reservoirs are affected by pore–throat blockage and mineral-related formation damage. Retarded autogenous mud acid has been applied to improve acid penetration and remove plugging while reducing rapid acid–rock reactions [19,20,21].

(2) Tight reservoirs

In contrast to the above low-permeability reservoirs, tight reservoirs represent a more restrictive reservoir category, characterized in this review by a permeability of <0.1 mD and a porosity of <10%. Under such extremely low-porosity and low-permeability conditions, oil and gas cannot flow effectively under natural conditions, and industrial-scale production generally requires artificial stimulation such as fracturing or acidizing. These restrictive petrophysical properties lead to severe acid-fluid leak-off, short effective penetration distances, and difficulties in spent-acid flowback [22]. For tight reservoirs, retarded acid systems can extend the effective penetration distance by reducing the acid–rock reaction rate and have demonstrated favorable application performance in the Sulige gas field [23,24,25]. Meanwhile, diverting and temporary plugging and diverting technologies can regulate acid-fluid migration using foam, gels, or degradable particles and are also applicable to tight reservoirs [26]. The Sulige Gas Field in the Ordos Basin is a representative tight sandstone gas reservoir. Its principal reservoirs occur in the Lower Shihezi and Shanxi formations and are characterized by strong compaction, heterogeneous fluvial sand bodies, poor pore connectivity, and low permeability. Acidizing and fracturing treatments are therefore used to improve reservoir connectivity and gas deliverability [27,28,29].

(3) High-temperature and high-pressure reservoirs

High-temperature and high-pressure (HTHP) reservoirs refer to oil and gas reservoirs with large burial depths and formation temperatures and pressures significantly higher than those of conventional reservoirs, featuring formation temperatures >120 °C and formation pressures >100 MPa. Such reservoirs are commonly found in offshore deep carbonate reservoirs, ultra-deep oil and gas reservoirs, and high-sour gas fields. They represent a crucial area for current oil and gas exploration and development and are extremely difficult to develop [30,31,32]. Under high-temperature conditions, the viscosity of the acidizing fluid drops significantly, and the leak-off volume increases significantly, making it difficult to achieve deep acidizing. High temperatures also accelerate the acid–rock reaction rate, causing the acid fluid to be consumed within a short time and shortening the effective penetration distance. Moreover, the stability of the acid fluid is poor, making it prone to demulsification or decomposition; at the same time, it exacerbates metal corrosion, imposing higher requirements on acidizing operation equipment and downhole tools [33,34]. The Shunbei Oilfield in the Tarim Basin provides an example of deep to ultra-deep carbonate reservoirs developed under high-temperature and high-pressure conditions. The reservoirs are dominated by complex fracture–vug systems, where rapid acid consumption at elevated temperature limits effective penetration. Retarded and in situ generated acid systems have therefore been investigated for deep acid-fracturing stimulation [35]. Strongly heterogeneous carbonate reservoirs provide another typical application scenario. Large permeability contrasts among layers cause conventional acid to preferentially enter high-permeability intervals, leaving tighter zones insufficiently stimulated. Field and experimental studies have therefore employed self-diverting or staged acid systems to redistribute acid toward poorly stimulated intervals and improve treatment uniformity [36,37,38,39].

(4) Strongly heterogeneous reservoirs

Strongly heterogeneous reservoirs refer to reservoirs with uneven permeability distribution and large differences in physical properties, mainly including strongly heterogeneous carbonate reservoirs, commingled production well reservoirs, and fracture-matrix coupled reservoirs [40]. In strongly heterogeneous reservoirs, acid fluids tend to advance along high-permeability dominant channels, making it difficult for the acid fluid to distribute evenly in low-permeability zones, resulting in poor stimulation effects [41]. This distribution pattern results in a limited effect on improving permeability in the low-permeability zones. In fracture-matrix coupled reservoirs, the interaction between natural fractures and artificial fractures is complex and difficult to predict. Meanwhile, it is highly difficult to control acid fluid diversion and routing, making it hard to achieve uniform acidizing [42,43].

### 2.2. Commonalities and Characteristics of Acidizing in Complex Reservoirs

#### Characteristics of Complex Reservoirs

Based on the aforementioned review of complex reservoirs, these reservoirs share two most prominent common characteristics: poor physical properties and strong heterogeneity [44,45,46]. The acidizing processes applied to various types of reservoirs also differ, as shown in Table 1.

Table 2 presents the advantages of the proposed acid systems for reference.

In terms of adaptability, the optimal choices for low-permeability reservoirs are foamed acid and retarded acid, whereas for the stimulation of tight reservoirs, emulsified acid and atomized acid should be prioritized. Under high-temperature and high-pressure environments, reservoirs are highly adaptable to in situ generated acid, followed by retarded acid; while for strongly heterogeneous reservoirs, the optimal targeted technology is diverting acid. Different types of complex reservoirs have their specifically matched dominant acidizing technologies. In practical engineering applications, precise technology selection must be conducted based on the core geological characteristics of the reservoir. The selection of stimulation method depends on reservoir permeability, formation damage, lithology, and the required stimulation extent. Matrix acidizing is generally conducted below the formation fracture pressure and is suitable for removing near-wellbore damage or improving matrix permeability. Hydraulic fracturing is preferred for tight or very low-permeability reservoirs when long conductive fractures are required. In carbonate reservoirs, acidizing can be combined with hydraulic fracturing as acid fracturing, in which acid is injected above the fracture pressure to create and etch fracture surfaces, thereby maintaining fracture conductivity after fracture closure [47]. Acid placement methods should also be selected according to well geometry and reservoir heterogeneity. Coiled tubing (CT) is particularly useful in long horizontal wells or heterogeneous intervals requiring selective acid placement, whereas conventional bullheading is simpler and more economical for relatively short and homogeneous intervals. However, CT may be limited by higher friction pressure and lower achievable injection rates [48].

**Figure 3:**
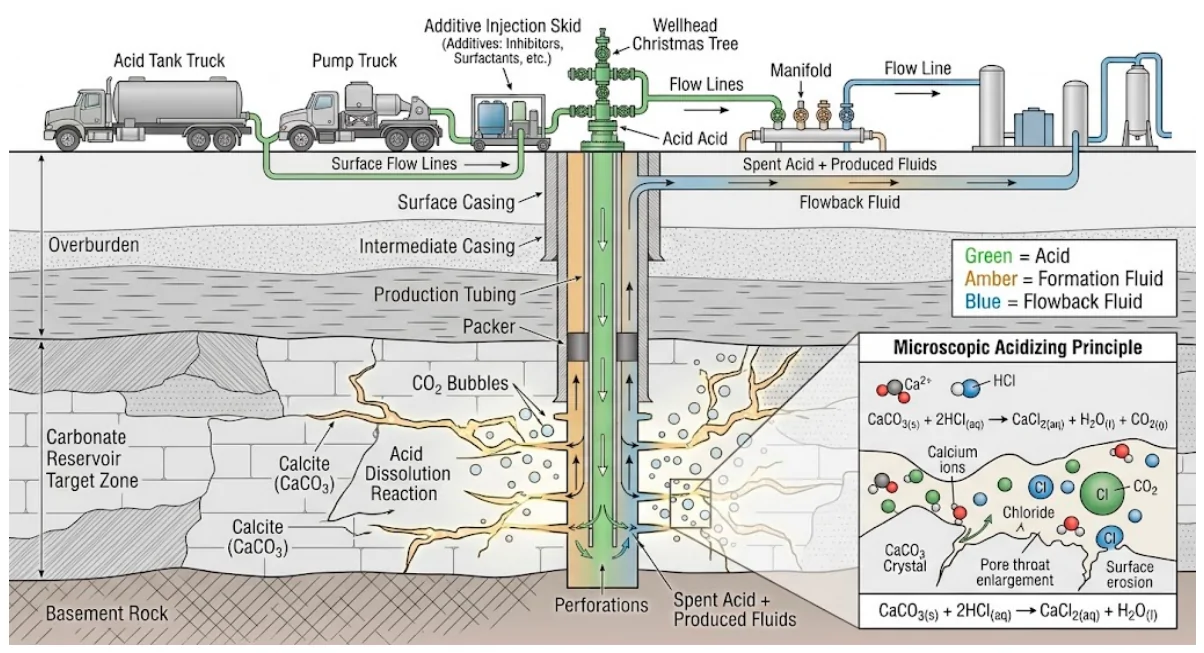
Acidification Process Technology Diagram.

A typical acidizing treatment consists of several sequential stages. First, reservoir mineralogy, permeability, formation damage, temperature, pressure, and fluid compatibility are evaluated to determine the appropriate acid formulation, additives, injection volume, and pumping rate. After well preparation, a preflush is commonly injected to condition the formation and minimize undesirable reactions between the main acid and formation fluids or minerals. The main acid is then pumped and placed into the target interval, where acid–rock reactions dissolve susceptible minerals, remove formation damage, and/or create conductive flow channels. Depending on the stimulation objective, matrix acidizing is conducted below the formation fracture pressure, whereas acid fracturing involves acid injection above the fracture pressure to create and etch fractures. After acid placement, a displacement fluid is used to push the remaining acid into the target zone, followed by well shut-in when required. Finally, the well is opened for flowback to recover spent acid, reaction products, and formation fluids before production is resumed. The overall acidizing process is illustrated in Figure 3 [49]. 

## 3. Research Progress on Acidizing Technology Systems

Before discussing the individual acidizing systems, it is necessary to distinguish the applicability of the principal acid chemistries to different reservoir mineralogies. HCl is primarily used for carbonate formations because it reacts readily with carbonate minerals such as calcite and dolomite. In contrast, HCl alone has limited ability to dissolve silicate and aluminosilicate minerals. HF-containing systems are therefore mainly used for sandstone and other silicate-rich formations, where HF can dissolve quartz, feldspars, and clay minerals. In sandstone acidizing, HF is commonly combined with HCl as mud acid; HCl primarily removes carbonate minerals and helps maintain favorable conditions for HF reactions, whereas HF provides the principal dissolution of siliceous and aluminosilicate components. Importantly, the six systems discussed in this review—foamed acid, retarded acid, emulsified acid, atomized acid, in situ generated acid, and diverting acid—represent functional formulation or delivery strategies rather than independent base-acid chemistries. Depending on reservoir mineralogy and stimulation objectives, these systems can be formulated using HCl, HF-containing acid mixtures, or other acid-generating chemistries. Therefore, their applicability should be evaluated jointly in terms of both the acid-system function and the mineralogical composition of the target formation [50,51,52,53]. 

For carbonate reservoirs with near-wellbore asphaltene deposition, HCl acidizing may temporarily improve productivity by creating conductive flow channels and bypassing the damaged region. However, contact between HCl and asphaltenic crude oil may induce acid–oil emulsion and asphaltic sludge formation, potentially causing secondary formation damage and limiting the long-term stimulation effect [54,55].

**Figure 4:**
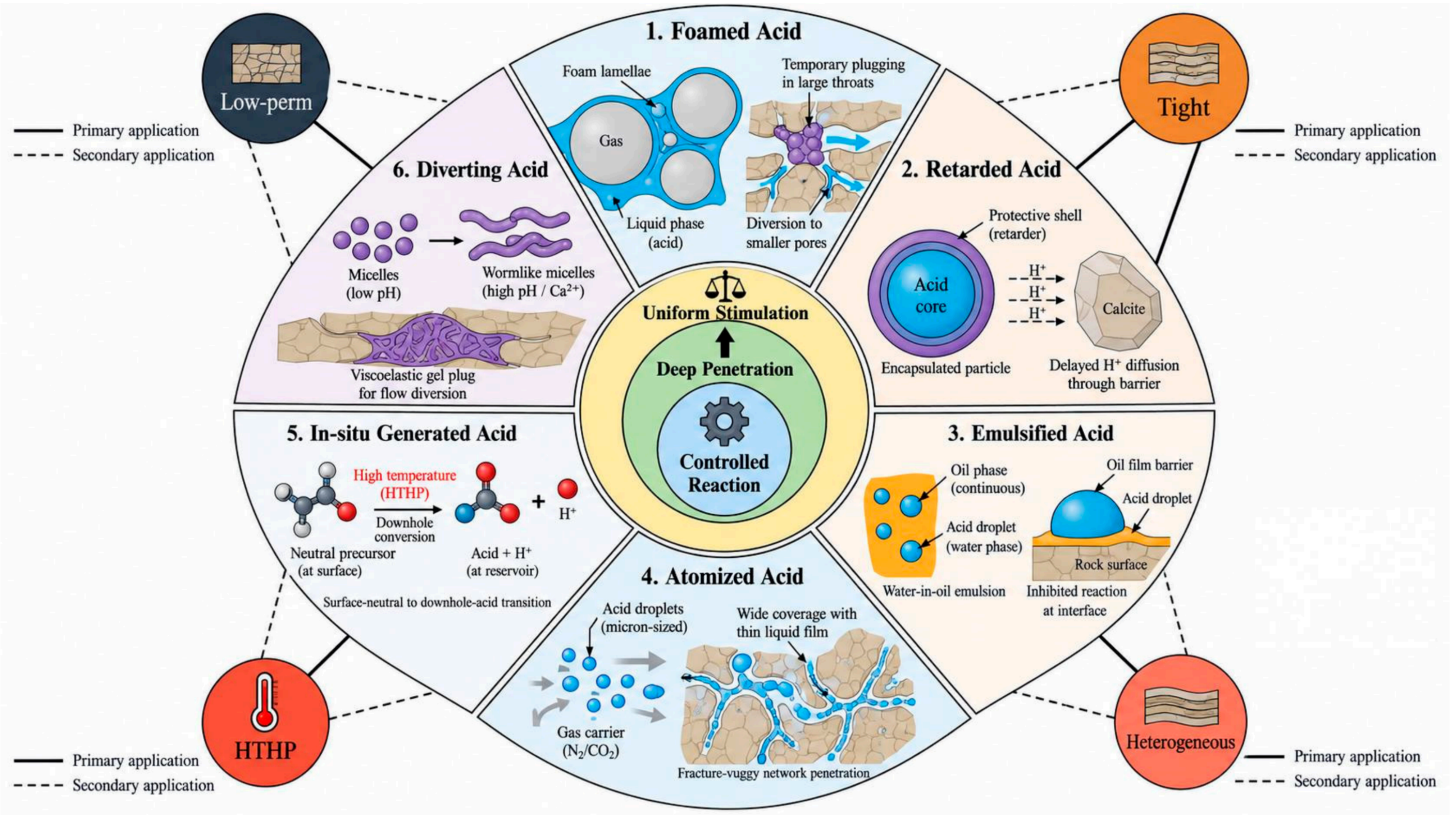
Typical interactions between six acidizing systems and reservoir formations.

To provide an intuitive overview of the acidizing systems discussed in this section, Figure 4 schematically illustrates the characteristic interactions of six representative acid systems with reservoir formations.

### 3.1. Foamed Acid System

Foamed acid systems are multiphase fluids constructed with an acid solution as the continuous phase, supplemented by foaming agents and foam stabilizers. As the mainstream technology to overcome the problem of acid channeling in heterogeneous reservoirs, this system integrates multiple functions, including temporary plugging and diverting, retarded acidizing, and reservoir protection [56].

In recent years, research on foamed acids has gradually evolved from single-formulation development toward multi-component synergistic regulation. In terms of flow mechanisms, the seepage characteristics of the gas–liquid two phases in porous media and their impact on reservoir stimulation have become research hotspots. Microscopic physical simulations indicate that foam undergoes a dynamic “collapse-regeneration” process within pores. This phenomenon can significantly adjust fluid migration paths and effectively improve the sweep volume of the acidizing fluid in heterogeneous reservoirs [57].

In terms of system optimization and regulation, through the synergistic enhancement of foaming agents and foam stabilizers or the introduction of novel functional materials, the temperature tolerance, salinity tolerance, and stability of foamed acids have been significantly improved [58]. This improvement in stability not only enhances the selective diverting capability of the system under different permeability contrasts but also further inhibits the acid–rock reaction rate, creating conditions for achieving deep retarded acidizing and effective etching in low-permeability reservoirs [59,60]. Relying on excellent comprehensive performance in foaming capability, reaction kinetics regulation, assisted flowback, and corrosion inhibition [61,62,63], the foamed acid system has become an important technical foundation for the highly efficient development of complex heterogeneous reservoirs.

### 3.2. Retarded Acid System

Retarded acid is an acidizing fluid system that delays the acid–rock reaction and extends the effective penetration distance of the acid fluid through special formulations and mechanisms. It is aimed at solving problems associated with conventional acids, such as rapid reaction rates, shallow penetration, and difficulty in spent acid flowback. It mainly includes types such as multi-hydrogen acids, encapsulated acids, and biological controlled-release acids, and is widely applied in deep plugging removal in low-permeability reservoirs.

In terms of plugging removal for water injection wells in low-permeability sandstone reservoirs, a retarded acid system with an optimized formulation—centered on a retarder and combined with corrosion inhibitors and flowback aids—possesses high retarding efficiency and good overall performance. In natural core experiments, it can significantly improve permeability, demonstrating a good plugging removal effect [64].

Retarded acid systems are widely applied in various types of complex reservoirs and serve as a key technology system to address the core pain points of deep reservoir acidizing stimulation. The research, development, and application of retarded acids have three core objectives [65,66,67,68,69]: delaying the reaction, deep penetration, and high adaptability. Aiming at bottlenecks such as the rapid reaction rate, short effective penetration distance, and difficult spent acid flowback of conventional acid fluids in high-temperature, high-stress, and strongly heterogeneous reservoirs, researchers at home and abroad have conducted systematic studies on retarding mechanism innovation, formulation system optimization, and performance regulation technologies. They have gradually formed a multi-type, multi-mechanism retarded acid technology system, providing core support for highly efficient plugging removal in deep reservoirs [70,71].

### 3.3. Emulsified Acid System

Emulsified acid is a water-in-oil (W/O) or oil-in-water (O/W) type acid fluid system formed by the emulsification of an acid phase and an oil phase under the action of surfactants. This system utilizes the shielding effect of the emulsion film to reduce direct contact between the acid fluid and the rock, thereby retarding the acid–rock reaction rate and extending the effective penetration distance [72].

Emulsified acid can promote an increase in the proportion of macropores in the pore structure and optimize the pore size distribution, improving reservoir flow characteristics and enhancing oil and gas productivity. Emulsifier concentration and droplet size have a significant impact on the acid diffusion capacity. As the emulsifier concentration increases, the emulsified droplet size decreases, the acid diffusion coefficient decreases, and the acid–rock reaction rate slows down accordingly [73,74,75].

Aiming at the problems of rapid acid–rock reaction rates and difficult-to-control tubular corrosion under high-temperature conditions, emulsifier systems have been further optimized. Within a suitable concentration range, an increase in emulsifier content can effectively reduce the acid–rock reaction rate and the acid diffusion rate. This is conducive to forming deeper wormhole structures in the reservoir while reducing the occurrence of face dissolution [76].

Addressing the bottlenecks of traditional emulsified acids, such as being prone to demulsification and causing formation damage due to high viscosity, researchers at home and abroad have focused on emulsion system optimization, polymer-free formulation innovation, and reaction kinetics regulation, forming multi-mechanism adaptable systems [77,78,79,80,81,82]. Studies show that optimizing emulsifiers or constructing low-viscosity systems can improve thermal stability and pumpability, significantly delay reactions, form deep etched channels, reduce acid consumption, and enhance stimulation effects, providing key support for the acidizing of high-temperature deep reservoirs [83,84,85]. 

### 3.4. Atomized Acid System

Atomized acid is a gas–liquid two-phase acidizing system formed by atomizing acid fluid into micron-sized droplets. Relying on the characteristics of gas–liquid two-phase flow and the liquid film shielding effect, it retards the acid–rock reaction and expands the action range. It can resolve the pain points associated with conventional acid fluids in high-temperature and high-pressure fractured-vuggy reservoirs, such as excessively rapid reactions, difficulty in achieving deep communication, and spent acid retention, and is widely applied in the acidizing stimulation of deep reservoirs, such as fractured-vuggy oil reservoirs developed by gas injection.

Focusing on the engineering application requirements of atomized acid systems, Zhang J et al. [86] proposed a new atomized acid acidizing process based on field practices in the Tahe Oilfield. Through laboratory experiments, they optimized the formulation and screened the optimal injection and production scheme, laying a theoretical foundation for field implementation. Under high-temperature conditions, the acid–rock reaction rate of atomized acid is significantly lower than that of conventional acid, and the corrosion rate is also reduced, which is conducive to forming effective communication channels in fractured-vuggy reservoirs and expanding the acidizing stimulation range.

The gas-acid system formed by combining acidic gas and acid fluid is also utilized to enhance the reservoir stimulation capability of atomized acid. A reasonable acid proportion can improve the migration and reaction characteristics of the acid fluid within the reservoir and enhance the permeability enhancement effect in tight sandstone reservoirs, demonstrating strong application potential. The gas–acid system studied by Bai et al. [87] also falls into this category.

### 3.5. In Situ Generated Acid System

In situ generated acid technology utilizes precursors such as esters, anhydrides, ammonium salts, halides, and carbonyl compounds to undergo chemical reactions and generate acidic substances under formation temperature conditions. This type of system is typically in a neutral or weakly acidic state under surface conditions, while gradually releasing acid in the high-temperature subsurface environment to achieve a delayed acidizing effect. It has become an important development direction in the field of acidizing stimulation for high-temperature deep oil and gas reservoirs in recent years.

Studies have shown that the controllable-concentration in situ generated hydrochloric acid system can generate hydrochloric acid within a stable concentration range under formation conditions, and still maintain good dissolution capability in a high-temperature environment of 180 °C, providing a new technical option for the acidizing of deep carbonate reservoirs [88]. For higher temperature conditions, the composite in situ generated acid system is even better, extending the temperature tolerance range to approximately 200 °C. It possesses strong acid-generating capability and can meet the requirements of acidizing operations in ultra-deep wells [89]. In addition, the in situ generated mud acid system exhibits good retarding and long-lasting characteristics in deep reservoir plugging removal. Under continuous action conditions, it can effectively clear deep plugged channels and improve reservoir seepage capacity.

To optimize the temperature tolerance, acid generation rate regulation, and acid–rock reaction behavior of in situ generated acids, related studies have optimized the reactant ratio and system composition. This can effectively extend the duration of acid generation, allowing the system to maintain a slow acid release state for a longer period at 180 °C, thereby improving the reservoir stimulation effect [90]. Meanwhile, through the optimization of the related reaction system ratios, the reaction rate can be reduced while extending the acidizing action time, maintaining good conductivity in acid-etched channels [91].

Currently, in situ generated acid technology has been tested and widely applied in multiple oil and gas fields [35,92,93]. This technology can significantly increase oil well production and demonstrates excellent stimulation effects in the acidizing of deep carbonate reservoirs. Its application has gradually become an important technical means to improve development effectiveness in some deep oil and gas fields [94,95]. At the same time, related studies have systematically summarized the development history and application experience of in situ generated acid technology, providing an important reference for subsequent system optimization and field promotion [96].

### 3.6. Diverting Acid System

Diverting acid systems achieve automatic diversion and uniform acid placement during injection by introducing functional components such as viscoelastic surfactants (VES), temporary plugging particles, fibers, or foam. This system can preferentially plug high-permeability channels, forcing the subsequent acid fluid to divert into low-permeability zones, thereby achieving uniform stimulation across the entire wellbore and all intervals [97].

Viscoelastic surfactant (VES) self-diverting acid is currently the most maturely applied system. Its core mechanism lies in utilizing the chemical environment changes triggered by H^+^ consumption and the increase in Ca^2+^ concentration during the acid–rock reaction [98] to drive VES molecules to self-assemble and form a wormlike micelle network. This causes the viscosity of the spent acid to increase exponentially, thereby dynamically plugging high-permeability channels and achieving the diversion of the acid fluid into low-permeability zones [99]. Under high-temperature and complex operating conditions, through the design of acid-sensitive and calcium-sensitive dual-responsive surfactants, the temperature tolerance of the VES system can be elevated to 120 °C or even higher. Meanwhile, further optimized high-temperature-resistant VES systems can adapt to extreme environments of 160 °C, significantly expanding the application boundaries of the technology [100,101]. Optimizing acid placement parameters according to reservoir characteristics can achieve significant production enhancement effects [102,103].

To further enhance diversion accuracy and improve spent acid flowback capability, composite diversion and residue-free technologies are developing rapidly. The system coupling degradable fibers with VES self-diverting acid can achieve more thorough acid fluid diversion in extremely heterogeneous reservoirs, avoiding the limitations of a single VES system under highly heterogeneous conditions [104]. CO_2_ foamed diverting acid utilizes the interfacial effects of gas in porous media to achieve dynamic diversion, providing a new, eco-friendly, and highly efficient approach with no solid residue for carbonate reservoir acidizing [105].

After diversion, effective viscosity reduction is essential for spent-acid cleanup and flowback. For polymer-based systems, viscosity can be reduced through pH-responsive gel breaking or chemical breakers. In VES-based systems, contact with reservoir hydrocarbons can disrupt wormlike micelles and substantially decrease fluid viscosity. Internal breakers can also be incorporated to provide controlled viscosity reduction, particularly when hydrocarbon contact alone is insufficient. These viscosity-reduction mechanisms facilitate fluid flowback and reduce the risk of secondary formation damage.

Field practices indicate that self-diverting acids and their composite systems possess good balanced production enhancement effects in multi-layer reservoirs and complex types of reservoirs, which can significantly overcome the problem of interlayer interference [106]. In offshore reservoir applications, high-performance diverting acid systems can simultaneously meet the requirements of high-temperature resistance and seawater compatibility, and the magnitude of production increase can exceed three times [107,108].

In addition to conventional acid formulations, functional additives such as nanoparticles and chelating agents have attracted increasing attention in acidizing treatments. Nanoparticles can be incorporated into acid systems to improve fluid stability and acid diversion, thereby enhancing acid placement in heterogeneous formations. Chelating agents, including EDTA, HEDTA, DTPA, and GLDA, can complex metal ions, reduce secondary precipitation, and provide more controlled mineral dissolution. Owing to their relatively low corrosion and slower reaction characteristics, some chelating agents can also be used as alternative stimulation fluids for carbonate and sandstone reservoirs, particularly under high-temperature conditions [109,110,111,112].

## 4. Application of Molecular Technology in Acidizing Technology Optimization

To break through the technical bottlenecks of traditional acidizing stimulation and realize the development of acidizing processes toward controllability, precision, and low damage, molecular simulation technology based on microscopic interaction mechanisms has emerged. By fundamentally optimizing the comprehensive performance of acid fluids through molecular structure modification and precise interfacial regulation, it effectively makes up for the defects of traditional acidizing, and has now become a cutting-edge research focus in the field of oil and gas reservoir stimulation.

**Figure 5:**
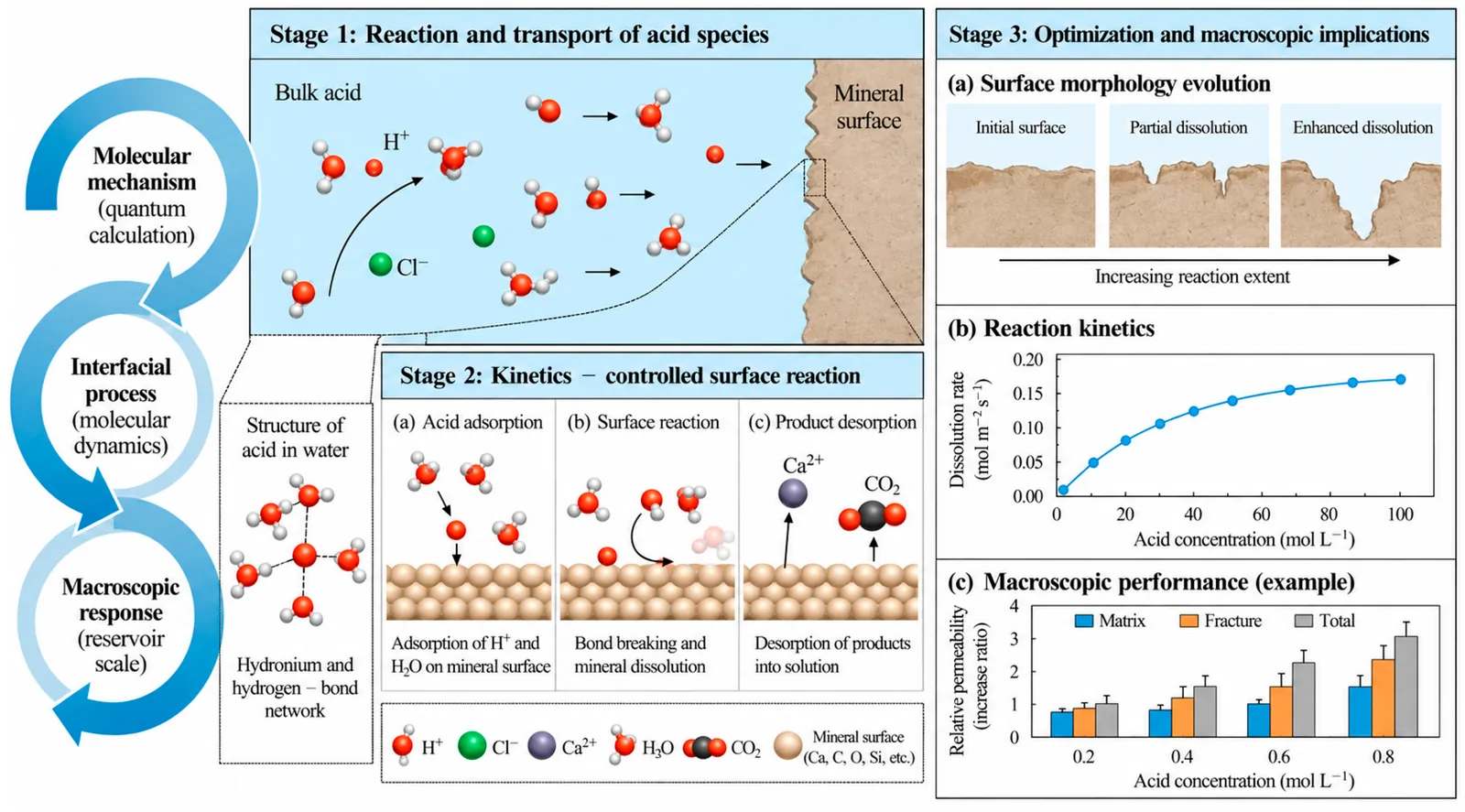
Application of Molecular Technology.

Molecular simulation uses computers to construct molecular models, which can both observe molecular structures and simulate the dynamic changes in molecules. It generally has three applications: first, analyzing experimental phenomena to reveal the underlying physical and chemical reaction mechanisms; second, achieving the targeted optimization of reagent formulations based on theoretical predictions; and third, systematically optimizing fluid injection parameters to enhance overall engineering effectiveness, as shown in Figure 5 [113].

Currently, two simulation methods are commonly used in the field of reservoir acidizing:

1. Molecular dynamics (MD): It calculates atomic motions based on classical mechanics equations to intuitively represent the processes of flow, adsorption, and corrosion among molecules.

2. Quantum chemical calculations: It analyzes reactions based on electronic-level equations and can calculate the energy, reaction difficulty, and reaction pathways of acid–rock reactions.

For the application of molecular technologies in the acidizing field, they are primarily applied in acid–rock reactions, formulation optimization, and injection condition optimization. The optimization of traditional reservoir acidizing technologies typically relies on laboratory experiments and field trial-and-error, which brings a certain degree of blindness to the research and development of acid fluid systems and the design of process parameters.

Molecular simulations of acid–rock systems are generally conducted using software packages such as Materials Studio, LAMMPS, GROMACS, and VASP. Materials Studio provides integrated modules for molecular modeling, Monte Carlo calculations, molecular dynamics, and quantum-mechanical calculations, whereas LAMMPS and GROMACS are widely used for large-scale classical molecular dynamics simulations. VASP is mainly employed for first-principles calculations based on density functional theory (DFT). In classical MD simulations, atomic trajectories are obtained by numerically integrating Newton’s equations of motion, while intermolecular interactions are described using appropriate force fields. Periodic boundary conditions are commonly applied to represent bulk or interfacial systems, and temperature and pressure are controlled using thermostats and barostats. Long-range electrostatic interactions can be treated using methods such as Ewald summation or particle–particle–particle–mesh (PPPM). For systems involving bond breaking and formation during acid–mineral reactions, reactive force fields such as ReaxFF can be employed to describe chemical reactions dynamically. DFT calculations are mainly used to determine adsorption energies, reaction pathways, electronic structures, and activation barriers at mineral surfaces. Table 3 lists commonly used molecular simulation techniques and software tools.

**Figure 6:**
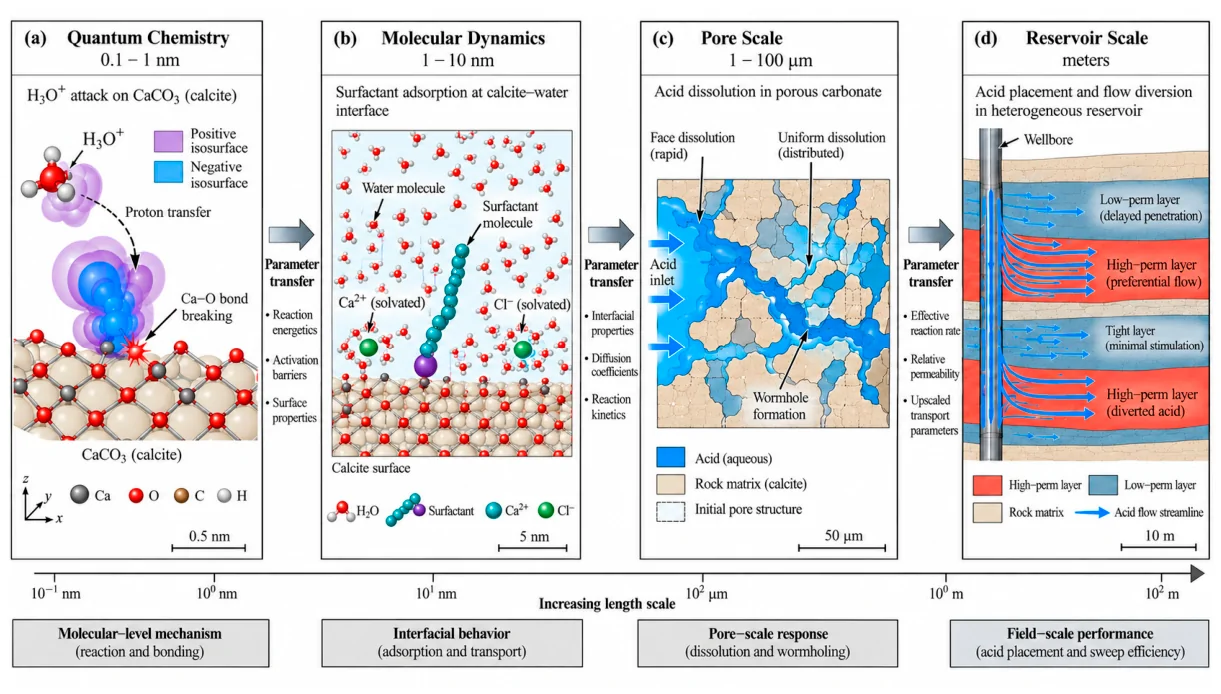
Schematic illustration of the multiscale numerical framework for acidizing.

Acidizing is inherently a multiscale process involving physicochemical phenomena ranging from molecular-scale acid–mineral reactions to field-scale acid transport and placement. As illustrated in Figure 6, different numerical approaches are therefore required to characterize acidizing behavior across multiple spatial scales. Quantum chemical calculations provide fundamental information on reaction pathways and energy barriers, while molecular dynamics simulations describe interfacial adsorption and molecular transport. These molecular-level parameters can further support pore-scale simulations of acid dissolution and wormhole development and, ultimately, reservoir-scale modeling of acid placement and flow diversion. Such a multiscale framework provides a bridge between fundamental acid–rock reaction mechanisms and field-scale stimulation performance.

### 4.1. Study on Acid–Rock Reaction Mechanisms

The acid–rock reaction mechanism is the core theoretical foundation of acidizing technology. Macroscopic regularities such as reaction rates, dissolution morphologies, and changes in rock mechanics are essentially determined by microscopic processes at the molecular scale, including chemical bond cleavage, ion migration, and interfacial adsorption. Therefore, dynamic behaviors at the molecular level can quantitatively reveal the microscopic pathways and action mechanisms of acid–rock reactions.

The core of carbonate reservoir acidizing is the reaction between H^+^ and carbonate minerals. Researchers have employed ab initio molecular dynamics combined with multi-scale quantum mechanics methods to analyze the formation mechanism of HCO_3_^−^ in alkaline/high-pH environments, which revised the traditional understanding of reaction pathways [114,115]. In addition to the simple proton transfer process, reactions such as mineral dissolution and interfacial oxidation-reduction in acid fluid systems are often accompanied by the transfer of protons and electrons. Studies on the proton-coupled electron transfer mechanism indicate that electron acceptors and proton acceptors can act synergistically to complete the activation process of the reaction, a mechanism that has been verified in related studies [116].

Targeting the fingering phenomenon during the injection process, the core solution is to retard the mass transfer and reaction of H^+^ by introducing additives. By combining DFT calculations and MD simulations, related scholars systematically revealed the inhibition rules of the carbon chain length of Gemini surfactants on acid–rock reactions from the perspectives of electronic structures and molecular adsorption. DFT electronic structure calculations demonstrate that surfactants with longer carbon chains have poorer compatibility with the calcite surface and are more likely to be distributed on the rock surface in a tilted configuration. MD simulations further confirmed from the perspective of molecular motion that an increase in carbon chain length exacerbates the aggregation effect of surfactant molecules, thereby reducing their adsorption density and effective coverage area on the rock surface [117].

Addressing the changes in rock mechanical properties caused by acid etching, researchers applied reactive molecular dynamics to simulate the stress corrosion cracking process of calcite in a carbonic environment. The study found that Ca-O bonds in the calcite crystal break preferentially, and H atoms from the liquid phase can form H-O bonds and C-H bonds on the rock surface. Among them, the H-O bonds bind with Ca atoms, further weakening the strength of the Ca-O bonds and accelerating their cleavage [118].

### 4.2. Optimization of Acid Formulation

Based on the understanding of the molecular mechanisms of acid–rock reactions, acid fluid systems with superior performance can be developed through molecular structure design, functional group regulation, and component compounding, which can be applied in multiple directions, including thickened acids, retarded acids, and corrosion inhibitor additives.

Targeting the defects of traditional cross-linked acids, which rely on cross-linking agents to increase viscosity and are prone to generating residues that cause secondary formation damage, researchers synthesized a non-cross-linked self-thickening polymer, ADDA. Calcium chloride generated during the acid–rock reaction increases the solution’s polarity, promotes the hydrophobic association of ADDA molecules, and forms a three-dimensional network structure. This allows the apparent viscosity of the acid fluid to increase gradually as the reaction proceeds, equipping the system with both excellent self-thickening capability and high-temperature resistance [119]. Furthermore, some scholars synthesized a temperature-resistant gelling agent through molecular structure optimization and compounded it with dodecyl betaine as a retarding synergist, successfully constructing a high-temperature-resistant retarded gelled acid system [120].

Corrosion of downhole tubulars and equipment by acid fluids is also a major risk during acidizing operations. Molecular simulation technology can similarly guide the molecular design and performance pre-evaluation of corrosion inhibitors. Addressing the corrosion of carbon steel in hydrochloric acid systems, various novel corrosion inhibitors, such as AEPA, DOCA, TMBN, and NTHA, have been developed and optimized through a combined approach of “experimental verification + DFT/MD simulations” [121,122,123]. The application of molecular technologies has enabled the prediction and optimization of corrosion inhibitor performance, significantly shortening the research and development cycle of new corrosion inhibitors.

### 4.3. Optimization of Acid Injection Conditions

Acid injection parameters directly determine the penetration depth, dissolution morphology, and ultimate stimulation effect of the acid fluid. Reaction mechanisms at the molecular and pore scales can provide theoretical support for optimizing injection conditions, achieving the matching of injection processes under different reservoir conditions.

The dissolution mode of acid fluids in reservoir pores is determined by the relative relationship between the reaction rate and the mass transfer rate, which can be characterized by the Damköhler number (Da, an indicator of reaction intensity) and the Péclet number (Pe, an indicator of convection intensity). Simulation studies show that under the conditions of high Pe and low Da, the acid fluid undergoes uniform dissolution within the pore structure, and the elastic modulus of the rock exhibits a slow and steady decline. Conversely, under the conditions of high Da and low Pe, face dissolution occurs at the injection end, causing a rapid and substantial decrease in the rock’s mechanical moduli. Furthermore, the attenuation of the shear modulus is faster than that of the bulk modulus, making the rock more susceptible to shear deformation [124]. Based on these principles, injection parameters can be appropriately adjusted during field operations.

The mechanical properties of carbonate rocks continuously deteriorate with prolonged acid etching time and increasing temperature [125]. By combining this with acid–rock reaction kinetic equations established based on molecular mechanisms, the reaction rates and activation energies under different temperatures and acid concentrations can be quantitatively calculated, providing a quantitative basis for the design of injection parameters.

## 5. Existing Challenges and Development Trends of Acidizing Technology

### 5.1. Existing Challenges

Although technologies such as foamed acid, retarded acid, emulsified acid, atomized acid, in situ generated acid, and diverting acid have been widely applied in the stimulation of complex reservoirs, given that oil and gas exploration is extending to deep, ultra-deep, and strongly heterogeneous reservoirs, we believe that existing systems still face severe challenges in terms of adaptability to extreme working conditions, diversion control accuracy, dissolution process characterization, and environmental friendliness [126].

(1) Fluid instability under extreme reservoir conditions

Under deep high-temperature reservoirs (exceeding 150 °C or even above 200 °C), acid fluid systems are prone to instability and failure [127]. Tubular corrosion is exacerbated, and existing corrosion inhibitors lack long-term protection, restricting the utilization of deep reservoirs [128]. High temperatures intensify molecular thermal motion, which not only weakens surfactant adsorption in multiphase systems, triggering phase separation, but also synergizes with shear to cause polymer chain scission and viscosity reduction, leading to acid fluid fingering and leak-off [129]. Meanwhile, the sudden increase in the acid–rock reaction rate causes massive consumption of the acid fluid near the wellbore, and corrosion inhibitors are also prone to thermal desorption and molecular degradation, losing their protective effects. The anti-corrosion issue has become a core bottleneck in deep acidizing [130].

(2) Deep mass transfer in heterogeneous porous media

Current acidizing operations mostly rely on empirical presets, making it difficult to adjust diversion strategies in real time according to downhole conditions; thus, uniform stimulation across the entire wellbore is hard to achieve. Poor spent acid flowback easily induces secondary formation damage, and polymers and solid temporary plugging agents are prone to plugging pore throats in low-permeability tight reservoirs. Moreover, traditional chemical additives have poor biodegradability, and the high treatment cost of flowback fluids does not meet the requirements of green and low-carbon development [131]. In fractured-vuggy carbonate rocks and multi-layered sandstone reservoirs, the permeability contrast is large, and existing diverting acid systems struggle to balance plugging strength and operation pressure: if the plugging strength is insufficient, the acid fluid easily channels into high-permeability streaks, leading to inadequate stimulation in low-permeability zones; if the plugging is too strong, the wellhead pressure rises sharply, which can easily trigger unintentional fracturing and lead to operational failure.

(3) Characterization and quantification of the dynamic acid etching process

On the experimental side, limited by size effects and extreme temperatures and pressures, traditional core experiments struggle to reproduce large-scale fluid-solid coupling processes. Although high-precision technologies such as Micro-CT can observe mineral and pore changes before and after the reaction, they cannot achieve high-resolution, non-destructive tracking of the transient acid etching process under high temperature and high pressure. On the numerical simulation side, constrained by multi-field, non-linear, multi-scale coupling, existing models struggle to achieve strict physical coupling between microscopic interfacial behaviors and macroscopic seepage and pore evolution. Furthermore, they mostly rely on empirical parameters and cannot accurately depict the non-uniform dissolution and fingering regularities of acid fluids in heterogeneous media.

(4) Scale connection difficulties between molecular-scale mechanism research and engineering field applications

Current molecular technology research in the field of acidizing is mostly based on ideal simplified models of pure mineral crystals, static reactions, and single-component acid fluids, which differ significantly from the real working conditions of deep complex reservoirs characterized by multi-mineral coexistence, high temperatures and high pressures, dynamic acid fluid flow, and the multi-field coupling of stress, seepage, and chemical reactions. At the same time, microscopic mechanistic conclusions at the molecular level have not yet formed engineering methods that can directly and quantitatively guide the debugging of acid fluid formulations and the optimization of injection parameters. The degree of matching between simulation prediction results and actual field stimulation effects still needs improvement. An obvious scale gap exists between microscopic mechanism research and macroscopic engineering applications, restricting the direct guiding efficacy of molecular technologies on acidizing operations.

### 5.2. Development Trends

In addition to conventional acidizing, several alternative stimulation techniques can be selected according to reservoir properties and formation-damage mechanisms. Hydraulic fracturing improves reservoir connectivity by creating conductive fractures and is particularly suitable for tight and ultra-low-permeability reservoirs [132,133]. Chelating agents, such as EDTA, HEDTA, and GLDA, can provide controlled mineral dissolution with lower corrosion and reduced secondary precipitation and may partially replace conventional strong acids under high-temperature conditions. Solvent or surfactant treatments are mainly applicable to organic formation damage, such as asphaltene or paraffin deposition, whereas thermal stimulation can improve fluid mobility or induce thermally generated fractures in suitable reservoirs. The major advantages and limitations of these alternative techniques are summarized in Table 4. Additional options include residual-oil-targeted treatments and engineered brines; acoustic stimulation [134]; gas or foam injection [135]; microbial EOR [136,137,138]; refracturing, fracturing-flooding, and integrated unconventional-reservoir stimulation [139,140,141]; hydrogel/surfactant, triphilic-surfactant–CO_2_, and molecular-deposition-film treatments [142,143,144]; in situ CO_2_ and binary-mixture technologies [145,146]; and gas-assisted, multi-thermal-fluid, or combined steam processes for heavy oil [147,148,149,150]. Alternative carbonate fluids and geothermal acidization broaden the applicable treatment envelope [151,152]. A news-index summary also reports a gel-polymer system for harsh reservoir conditions and flow control [153].

**Figure 7:**
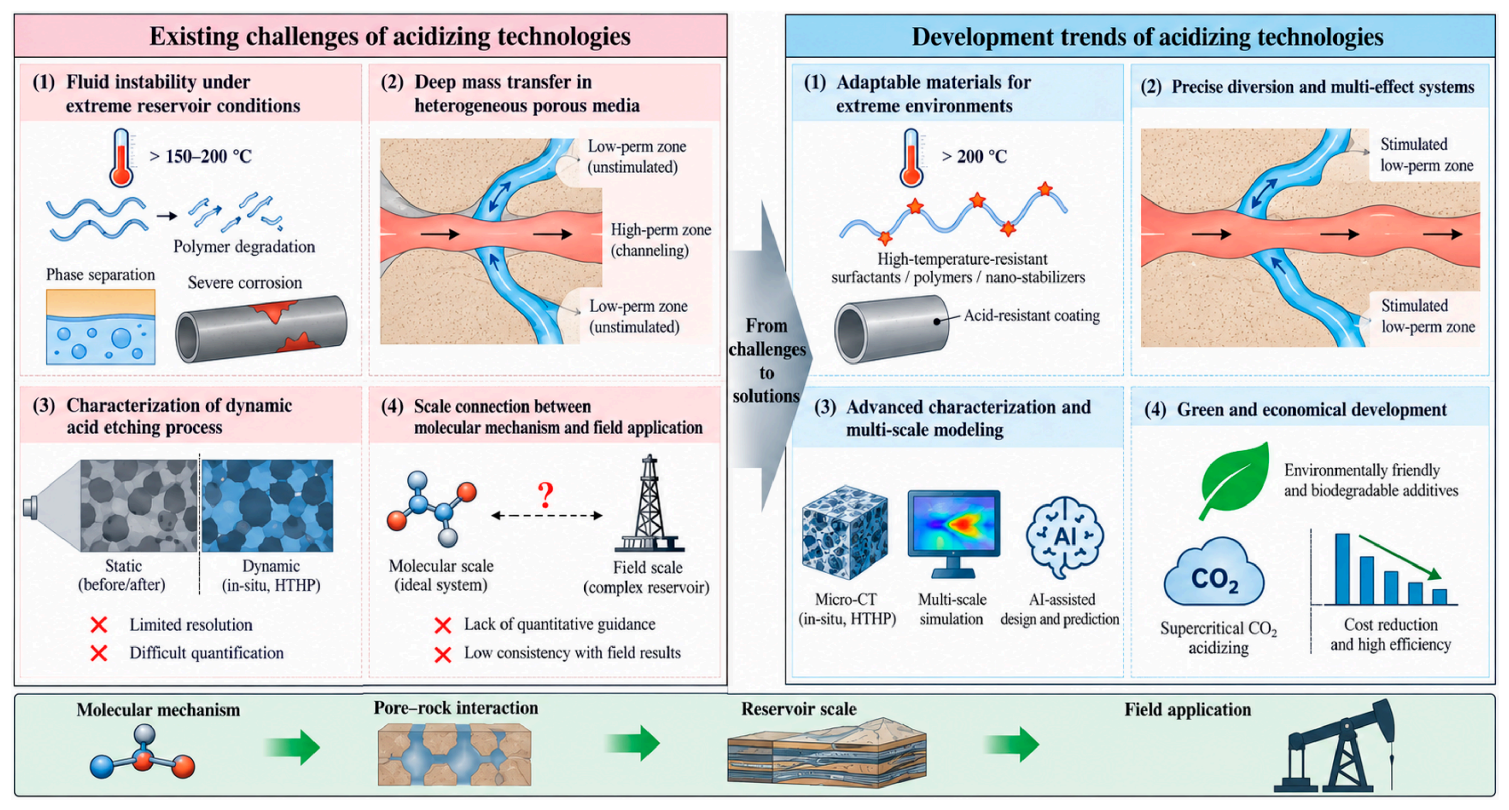
Challenges and Development Trends of Acidizing Technologies.

Targeting the existing problems of acidizing technologies, the future development of acidizing technologies needs to focus on breakthroughs in adaptable materials for extreme environments, including the adaptation of acid fluid systems and acid-resistant tubular coatings. The research and development of novel high-temperature-resistant surfactants, polymers, and nano-stabilizers that can withstand temperatures above 200 °C is also a core direction. Meanwhile, existing studies indicate that supercritical CO_2_-based acidizing technology, due to its unique phase characteristics and excellent mass transfer capabilities, will become an important supplement for solving the difficulty of retardation at high temperatures [154]. In terms of process mechanisms, multi-effect, refinement, intelligentization, and environmental friendliness will always be the major development directions. AI intelligence can be utilized and combined with molecular simulation technology to achieve delicate operations such as formulation optimization, real-time monitoring and decision-making, and model deduction. This greatly reduces costs while improving the pertinence, accuracy, and reliability of reservoir stimulation. Aiming at the technical bottlenecks in the stimulation process of deep and ultra-deep oil and gas reservoirs, Figure 7 comprehensively analyzes the existing challenges of current acidizing technologies and further points out the future research directions centered on the combination of macroscopic and microscopic mechanisms, the development of eco-friendly multi-functional systems, and targeted precise design [155,156].

Furthermore, a disconnect exists between laboratory research and field practice, with economic issues being the primary contradiction. Therefore, while developing novel technologies, research on reservoir acidizing stimulation technology must also focus on actual field applications, aiming to achieve both high efficiency and cost reduction.

## 6. Conclusions and Prospects

(1) Targeting the geological characteristics of complex reservoirs, such as poor physical properties and strong heterogeneity, acidizing working fluid systems including foamed acid, retarded acid, emulsified acid, atomized acid, in situ generated acid, and diverting acid have been developed. This has effectively achieved the adaptation between acid fluid systems and the stimulation requirements of multiple types of complex reservoirs.

(2) As oil and gas exploration and development accelerate their extension into deep and ultra-deep extreme geological environments, the limitations of existing technological systems have become increasingly prominent. Under harsh operating conditions such as ultra-high temperatures, high pressures, and strong heterogeneity, the thermodynamic instability of fluid materials, the limited efficiency of deep mass transfer, as well as the prevention of consequent secondary formation damage and high operational costs, remain severe challenges at present.

(3) At the level of basic theory and core material research and development, there is an urgent need to break down the barriers of single macroscopic models. Focusing on fluid instability, deep mass transfer, and fine quantitative characterization, advanced microscopic characterization methods such as molecular dynamics simulations, CT, and XRD should be utilized to depict the fluid-solid coupling dissolution mechanisms under extreme operating conditions.

(4) At the level of engineering design and field application, multi-functionality, refinement, intelligentization, and environmental friendliness will consistently serve as the core directions for industry development. It is urgently necessary to deeply integrate big data, numerical simulation, and artificial intelligence technologies to comprehensively enhance the targetability, precision, and reliability of extreme reservoir stimulation, on the basis of significantly reducing operational and environmental costs.

## Figures and Tables

**Table 1:** Development Challenges and Corresponding Acidizing Technologies for Various Complex Reservoirs.

Reservoir Type	Characteristics and Development Challenges	Corresponding Acidizing Technology
Low-permeability reservoirs	Low porosity and permeability, high clay mineral content, and poor fluid mobility; low productivity, prone to formation damage such as water blocking, emulsification, and fines migration.	Foamed acid, Retarded acid
Tight reservoirs	Nanoscale pores, ultra-low permeability, presence of organic matter and clay, unevenly developed natural fractures; difficult for acid to penetrate the matrix, requiring combination with fracturing for effective stimulation.	Emulsified acid, Atomized acid
HTHP reservoirs	Deep burial depth, extreme temperature and pressure conditions; excessively rapid acid–rock reaction rate, strong corrosivity, and premature acid spending.	In situ generated acid, Retarded acid
Highly heterogeneous reservoirs	Uneven permeability distribution and significant variations in petrophysical properties; acid preferentially advances through high-permeability channels, making uniform acid distribution difficult in low-permeability zones, leading to poor stimulation effectiveness.	Self-diverting acid

**Table 2:** Advantages of Acid Systems.

Acid System	Advantages	Limitations
Foamed Acid	Low fluid loss and strong carrying capacity, suitable for the stimulation of low-permeability/tight reservoirs; Foam diversion can improve uniform acid distribution in highly heterogeneous formations.	Poor temperature and salinity resistance, unstable properties; Complex preparation and quality control due to the use of multiple additives.
Retarded Acid	Reduces the acid–rock reaction rate and extends the effective penetration distance, suitable for HTHP reservoirs; Minimizes excessive near-wellbore dissolution.	Prone to degradation at ultra-high temperatures, leading to failure of the retardation effect; May generate residues, causing secondary formation damage.
Emulsified Acid	Excellent retardation effect and low fluid loss, capable of forming deep-penetrating and high-conductivity fractures; One of the preferred systems for acid fracturing in tight reservoirs.	Prone to demulsification at high temperatures, leading to functional failure; High viscosity and high friction loss.
Atomized Acid	Uniform acid dispersion and wide coverage, enhancing the sweep efficiency in highly heterogeneous reservoirs; Suitable for the fine stimulation of low-permeability reservoirs.	High equipment requirements and high operational costs; Narrow gas–liquid parameter window and unstable flow patterns.
In situ Generated Acid	Slowly generates acid within the formation, providing good retardation and fluid loss control; Achieves deep stimulation, particularly suitable for HTHP reservoirs.	Temperature-sensitive, which may shorten the penetration distance; Weaker acidity and relatively high cost.
Diverting Acid	Automatically increases viscosity in high-permeability zones for fluid diversion, achieving balanced stimulation of high- and low-permeability layers; Specifically suitable for highly heterogeneous reservoirs.	VES (viscoelastic surfactant) is prone to degradation at high temperatures, leading to diversion failure; Delayed viscosification results in poor early-stage diversion effects.

**Table 3:** Molecular simulation techniques.

Method	Typical Software	Main Application in Acidizing Studies
DFT	VASP, Materials Studio/CASTEP	Adsorption energy, reaction pathway, electronic structure
Classical MD	LAMMPS, GROMACS, Materials Studio	Diffusion, adsorption, transport, interfacial behavior
Reactive MD	LAMMPS/ReaxFF	Bond breaking/formation and acid–mineral reactions
Monte Carlo	Materials Studio	Molecular configuration, adsorption and equilibrium properties

**Table 4:** Advantages and disadvantages of alternative stimulation techniques to conventional acidizing.

Alternative Technique	Main Application	Advantages	Disadvantages
Hydraulic fracturing	Tight and ultra-low-permeability reservoirs	Creates long conductive fractures; large stimulation volume	High water and operational requirements; fracture propagation is difficult to control
Chelating-agent stimulation	Carbonate and sandstone reservoirs, particularly at high temperature	Lower corrosion; slower reaction; reduced secondary precipitation	Higher chemical cost; generally lower mineral-dissolution rate
Solvent/surfactant treatment	Organic damage, e.g., asphaltene and paraffin deposition	Effective removal of organic deposits; limited rock dissolution	Limited effectiveness for inorganic/mineral damage; chemical compatibility concerns
Thermal stimulation	Heavy-oil and temperature-sensitive reservoirs	Improves hydrocarbon mobility; may induce thermal fractures	High energy consumption; limited applicability and temperature-control difficulties

## Data Availability

No new data were created or analyzed in this study. Data sharing is not applicable to this article.
